# Phytochemicals in Leaves and Roots of Selected Kenyan Orange Fleshed Sweet Potato (OFSP) Varieties

**DOI:** 10.1155/2020/3567972

**Published:** 2020-01-28

**Authors:** George Ooko Abong', Tawanda Muzhingi, Michael Wandayi Okoth, Fredrick Ng'ang'a, Phillis E. Ochieng', Daniel Mahuga Mbogo, Derick Malavi, Machael Akhwale, Sita Ghimire

**Affiliations:** ^1^Department of Food Science, Nutrition and Technology, University of Nairobi, P.O. Box 29053-00625, Kangemi, Kenya; ^2^Biosciences Eastern and Central Africa-International Livestock Research Institute Hub, P.O. Box 30709-00100, Nairobi, Kenya; ^3^Food and Nutritional Evaluation Laboratory, International Potato Centre, P.O. Box 25171-00603, Nairobi, Kenya; ^4^Kenya Agricultural & Livestock Research Organization, KALRO Kakamega Centre, P.O. Box 169-50100, Kakamega, Kenya

## Abstract

This study reports the inherent phytochemical contents in leaves and roots of nine sweet potato varieties from Kenya. Results indicated that vitamin C content varied significantly (*P* < 0.05) among the sweet potato varieties regardless of the plant part, leaves having significantly (*P* < 0.05) higher levels than in the roots. Total flavonoids and phenolic compounds differed significantly (*P* < 0.05) among varieties, higher values were found in leaves than in roots. Flavonoid contents in roots ranged from below detectable limits (Whitesp) to 25.8 mg CE/100 g (SPK031), while in leaves it ranged from 4097 to 7316 mg CE/100 g in SPK4 and Kenspot 5, respectively. Phenolic content was below detectable limits in the roots of whitesp but it was in substantial amounts in orange fleshed varieties. The *β*-carotene content was significantly (*P* < 0.05) higher in leaves (16.43–34.47 mg/100 g dry weight) than in roots (not detected—11.1 mg/100 g dry weight). Total and phytic phosphorus were directly correlated with phytate contents in leaves and the roots. Tannins and soluble oxalates varied significantly (*P* < 0.05) with variety and plant part being higher in leaves. The current information is important for ration formulations and dietary recommendations utilizing sweet potato leaves and roots. Future studies on effects of processing methods on these phytochemicals are recommended.

## 1. Introduction

Sweet potato ranks seventh among the food crops in the world [1], and is a major contributor of energy and phytochemicals to the human diets, the extent of which depend on varieties and forms of utilization [2, 3]. For instance, the Orange fleshed sweet potato (OFSP) is a promising biofortified crop for sub-Saharan Africa (SSA) with high levels of *β*-carotene, a provitamin A carotenoid (pVAC) [4]. Biofortified OFSP has been proven to be affordable, convenient, and sustainable food source of pro-vitamin A carotenoids for combating vitamin A deficiency (VAD) in Kenya and other SSA countries [5, 6].

Apart from their high *β*-carotene content, OFSP varieties are known to have higher levels of other phytochemicals such as flavonoids, phenolics and anthocyanins [7] that may influence the quality and stability of processed products. These phytochemicals are known to enhance human health by acting antagonistically on incidences of cancers and chronic diseases, including cardiovascular disease (CVD), type II diabetes, and impaired cognitive function [8]. Due to their preventive effects against chronic diseases, they are considered as indispensable components in a variety of nutraceutical, pharmaceutical, medicinal and cosmetic applications [9, 10].

The levels of phytochemicals vary among the plant parts being high on leaves or colored roots [9]. Sweet potato leaves are consumed as vegetables in Islands of the Pacific Ocean, Asia, African countries, and to some extent in the United States of America [11]. These leaves contain both phytochemicals and antinutritional factors such as oxalates, tannins, and phytates, and the processing methods also influence their levels in food products [12]. Even though attempts have been made to determine phytochemicals content in sweet potato, the major focus has been on *β*-carotene with limited data on total phytochemicals and antioxidant activity variations among the OFSP varieties currently promoted in Kenya. Therefore, this study evaluated inherent phytochemicals in the leaves and roots of selected OFSP varieties in Kenya.

## 2. Materials and Methods

### 2.1. Acquisition of Sweet potato Leaves and Roots Samples

Seven OFSP and two popular white and yellow fleshed varieties namely; Kabode, Vitaa, Kenspot 5, Kenspot 4, SPK031, SPK004, and K/KA/2004/215, Whitesp and Yellowsp were grown at the Kenya Agricultural & Livestock Research Organization-Kakamega following the standard agronomic practices in 2017 season. Leaves and roots samples were harvested at maturity. The yellow and white fleshed sweet potato varieties were included as local check in the study. Important characteristics of test varieties are shown in Table 1. The leaves and roots were harvested, packaged in plastic net bags and gunny bags, respectively, and transported overnight to the International Livestock Research Institute (ILRI) for laboratory analysis.

### 2.2. Sample Preparation

Approximately 400 g clean sweet potato leaves of each variety was weighed and divided into portions of 100 g. The leaves were transferred to Kraft paper bags, frozen at −20°C for at least 12 hours and freeze dried (Telstar Lyoques-55, Spain). Similarly, seven roots were randomly selected for each variety, washed with tap water and blot dried, peeled, and diced into about 0.25 cm cubes. About 400 g of these cubes was divided into portions of 100 g, placed in Kraft paper bags, and frozen overnight at −20°C before freeze drying. Freeze-dried samples were ground using a warring laboratory electric blender into powder and stored at −20°C until analysis.

### 2.3. Analytical Methods

#### 2.3.1. Determination of Dry Matter Content

Moisture content of sweet potato leaves and roots was determined by forced air oven drying at 105°C as described by Abong' et al. [13].

#### 2.3.2. Determination of Vitamin C

Vitamin C in sweet potato leaves and roots was determined by HPLC as described by Fatariah et al. [14] with slight modification. Briefly, 2 g of fresh sample was weighed into 50 mL polypropylene tube and 30 mL of 3% metaphosphoric acid added and homogenized using a ProScientific homogenizer (ProScientific-200, USA). The mixture was sonicated in ultrasonic bath (Jircus BU-9500Z, Japan) for 5 min, vortexed and centrifuged at 845×*g* for 5 min. The supernatant was filtered using 0.25 *µ*m membrane for HPLC analysis. Sample separation was achieved using a Shimadzu UPLC system. Chromatographic separation was performed on a Shimadzu (Kyoto-Japan) Nexera X2 UPLC system equipped with a Shimadzu SIL-30AC auto-sampler, Shimadzu CTO-30A column oven, LC-30 AD pumps, and SPD-M20A Prominence Diode Array Detector. The analytical column used was C18 (Kinetex, 100 m × 3.0 mm, 2.6 *µ*m). The mobile phase composition consisted of 0.3 mM potassium dihydrogen phosphate in 0.35% (v/v) phosphoric acid at a flow rate of 0.2 mL/min at ambient temperature. Injections of 20 *µ*L were performed with a total run time of 12 min. Data were extracted at a wavelength of 242 nm; compound identification was based on matching of the retention times with pure ascorbic acid (Sigma Aldrich). Compound quantitation was carried out through external calibration using peak area method after integration of chromatographic peaks using Shimadzu LabSolutions software.

#### 2.3.3. Extraction of Phenolics and Flavonoids

Total phenolics and flavonoids in freeze dried OFSP roots and leaves were determined through colorimetric assay adopted to be used with a micro-titer plate and reader. Briefly, 0.15 g and 0.25 g of the freeze-dried leaf and root powder, respectively, were weighed into clean propylene tubes before addition of 10 mL of 80% methanol, vortexed (SI-0166, USA), and shaken on a mechanical shaker (Innova 43, USA) at 8×*g* and an incubation temperature of 25°C for 12 hours. The mixture was centrifuged at 3226×*g* for 10 min, and the supernatant aliquot was collected to determine the total phenolics and total flavonoid contents.

#### 2.3.4. Determination of Total Phenolics

The total phenolic content was determined using a modified Folin-Ciocalteu procedure [15]. Briefly, 20 *µ*L of the sample blank solution (80% methanol), gallic acid standards (10–100 *µ*g/mL) and samples were pipetted into their respective wells in a microtiter plate followed by addition of 100 *µ*L of 10% Folin–Ciocalteu phenol (Sigma Aldrich) reagent with gentle mixing by priming using a multichannel pipette. After 5 min, 80 *µ*L of 7% of sodium carbonate was added and primed gently before the plate was covered using an aluminum foil and the reaction left to incubate at room temperature for 90 min. Absorbance readings were obtained at 725 nm in a microtiter plate spectrophotometer reader (Synergy HT, USA). External standard calibration technique was used to quantify the concentration of total phenolic compounds in mg/100 g of the dry sample as Gallic Acid Equivalent (mg GAE).

#### 2.3.5. Determination of Total Flavonoids

The total flavonoid content was determined using aluminum chloride colorimetric procedure [16]. Briefly, 20 *µ*L catechin standards (10–100 *µ*g/mL) and samples were pipetted into to respective wells in a microtiter plate followed by addition of 80 *µ*L of deionized distilled water and 10 *µ*L of 5% sodium nitrite, and gently mixed by priming. After 5 min, 10 *µ*L of 10% aluminum chloride was added and primed gently before addition of 80 *µ*L of 2 M sodium hydroxide. The plate was covered with aluminum foil and the reaction left to proceed at room temperature for 30 min. Absorbance readings were obtained at 510 nm in a microtiter plate spectrophotometer reader (Synergy HT, USA). External standard calibration technique was used to quantify the concentration of total flavonoids in mg/100 g of the dry sample as Catechin Equivalent (mg CE).

#### 2.3.6. Determination of Carotenoids

 
*(1) Sample Extraction.* All sample preparation and sample analysis were conducted under yellow light to protect carotenoids from UV. The carotenoid analysis was performed according to a method described by Muzhingi et al. [17] with some modifications. Briefly, 0.5 g of freeze-dried powdered sample was mixed with 5 mL of absolute methanol and placed in a water bath (SW23GB, Germany) at 70°C for 10 min. The mixture was vortexed for 1 minute and centrifuged at 800×*g* (Eppendorf, Centrifuge 5810, Germany) for 10 min. Methanol layer was transferred into a 25 mL volumetric flask and subjected to extraction using 5 mL Tetrahydrofuran (THF), vortexed and centrifuged as previously described.

Extraction was repeated three more times using 5 mL of THF each time the supernatant layers being transferred into the volumetric flask. Methanol was added to make the final volume to 25 mL before mixing. To each 2 mL of the extract 0.5 mL of methanol, 4 mL of hexane, and 3 mL of HPLC water were added in a 25 mL glass tube. The mixture was vortexed for 1 minute and centrifuged at 800×*g* for 3 min. The upper phase was transferred into a 15 mL glass tube and dried completely under nitrogen gas using N-Evap machine (Organomation, Model OA-8125, USA) in a water bath set at a maximum of 40°C. The sample was reconstituted by addition of 2 mL of mixture of methanol and tetrahydrofuran (THF) (85 : 15 v/v) in a tube. The tube was then vortexed and sonicated (Jircus BU-9500Z, Japan) for 30 s before loading to HPLC vials.

 
*(2) Analysis of Specific Carotenoids.* Carotenoid analysis was carried out by use of HPLC (Waters 2695, USA) separation module with photo diode detector (Waters 2996, USA) using previously published methods [18]. The carotenoids were separated on a reverse phase C30column (YMC Wilmington, NC 150 × 4.6 mm, 3 *µ*m). The mobile phase composition consisted of eluent A being a mixture of methanol, tert-butyl methyl ether and 1.5% ammonium acetate in water (85 : 12:3, v/v/v) and eluent B being a mixture of methanol, tert-butyl methyl ether and 1% ammonium acetate in the water (8 : 90 : 2, v/v/v). A 40 min linear gradient elution programme was used and was set as follows: 0–1 min, 100% A; 1–10 min 100–90% A; 10–22 min 90–45% A; 22–33 min 45–5% A; 33–37 min 5% A; 37–39 min with a linear gradient to 5–100% A; 39–40 min 100% A. The injection volume was 40 *µ*L while oven temperature was set at ambient (25°C) and the carotenoids were monitored at a wavelength of 450 nm. All carotenoids in the samples were identified by comparing peak retention times and absorption spectra with that of known standards.

### 2.4. Determination of Antioxidant Activity

The total antioxidant activity of sweet potato leaves and roots was determined using 2,2 diphenyl-1-picrylhydrazyl (DPPH) procedure and the results expressed as Trolox equivalent. A concentration of 0.002% DPPH [19] was adopted with modification. Briefly 0.15 g and 0.25 g of freeze-dried powdered leaves and roots were respectively, weighed into 50 mL polypropylene tube and 10 mL of 80% methanol added before shaking in mechanical shaker for at least 12 hours (overnight). The mixture was centrifuged at 2588×*g* for 15 min and supernatant was used for analysis of antioxidant activity. Approximately 50 *µ*L of the blank, standards (5–50 *µ*g/mL Trolox) and samples were pipetted into their respective wells in a microtiter plate followed by addition of 50 *µ*L of 0.002% DPPH with gentle mixing by priming using a multichannel pipette. Absorbance reading was obtained at 515 nm in a microtiter plate spectrophotometer reader (Synergy HT, USA) within 10 min. A standard calibration curve of Trolox was used to calculate the concentration of total antioxidant activity in mg per 100 g of the dry sample and expressed as mg of Trolox Equivalent (mg TE).

### 2.5. Determination of Phytate and Phytic Phosphorus

Determination of phytates and phytic phosphorus was accomplished using a commercially available assay Kit, K-PHYT 11/15 (Megazyme International, Ireland) with slight modifications to fit microtiter plate reader as opposed to the low throughput conventional UV/VIS spectrophotometer. Sample extraction procedures were carried out as per the assay kit. However, enzymatic dephosphorylation reaction volumes were varied downwards with a factor of 5. This variation was also applied when pipetting samples into microtiter plate. Oat meal flour supplied with the kit was analyzed alongside the samples as a reference sample for quality control purposes. Reliable and reproducible results were obtained with the total phosphorus and phytic acid content variations being within 10% specified by the procedure.

### 2.6. Determination of Tannins

Tannins (tannic acid) in sweet potato leaves and roots were determined according to a method described by Saxena et al. [20]. Approximately 0.2 g and 0.15 g of freeze-dried powdered roots and leaves respectively were weighed into 250 mL conical flasks and 35 mL water added. The flask was heated gently and allowed to boil for 30 min. The resultant solution was transferred into 50 mL polypropylene tube and topped to 50 mL using deionized water and centrifuged at 1902×*g* for 10 min. The supernatant was collected into separate vials. Into a 96 well microtiter plate, 50 *µ*L of sample (supernatant), standards (tannic acid) and blank solution was added followed by addition of 50 *µ*L of Folin–Denis reagent and 100 *µ*L of 7% sodium carbonate solution before mixing by priming using multichannel pipette. The absorbance reading obtained at 700 nm after 30 min. A standard calibration curve of Tannic acid was used to calculate the concentration of total tannins in mg per 100 g of the dry sample.

### 2.7. Determination of Soluble Oxalates

Soluble oxalate extraction was carried out as per the procedure described by Nguyen and Savage [21], while chromatographic separation was accomplished based on Wang et al. [22] with minor modifications. For soluble oxalates, 0.5 g freeze-dried sample was weighed into polypropylene tube, 20 mL of deionized water was added with the resultant solution shaken for 15 min and centrifuged at 2588×*g* for 15 min. The supernatant was filtered through 0.45 nm cellulose nitrate filters into HPLC vials. The obtained extract was analyzed by HPLC. Chromatographic separation was performed on a Shimadzu (Kyoto-Japan) Nexera X2 UPLC system equipped with a Shimadzu SIL-30AC autosampler, Shimadzu CTO-30A column oven, LC-30 AD pumps and SPD-M20A Prominence Diode Array Detector. The analytical column used was C18 column (Kinetex, 100 m × 3.0 mm, 2.6 *µ*m). An isocratic gradient elution program was used using 0.02N sulfuric acid as the mobile phase at a flow rate of 0.2 mL/min and oven temperature set at 40°C. Injection volume of 20 *µ*L was used with a run time of 10 min. Oxalic acid standards were prepared for use in identification and quantitation through external calibration. 

## 3. Results and Discussion

### 3.1. Leaves and Roots Dry Matter Content

**Figure 1:**
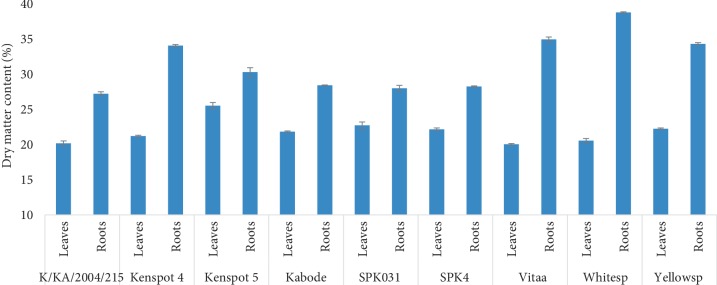
Variations in dry matter contents in leaves and roots of nine Kenyan sweet potato varieties. The bars indicate standard error of the means.

Dry matter content varied significantly (*p* < 0.05) among sweet potato varieties and plant part. The roots hard higher percent dry matter compared to leaves (Figure 1). The dry matter content ranged 27.21–38.78% and 20.05–25.53% in roots and leaves, respectively. With exception of Kenspot 4, Kenspot 5, and Vitaa that had root dry matter contents (above 30%) comparable to local check white and yellow varieties, other OFSP varieties had lower root dry matter contents. The dry matter contents reported in this study were comparable to those reported in a previous study [23]. For the processing purposes, varieties with low dry matter contents are undesirable since they give low yield and absorb more oils when fried products are produced from them [18].

Apart from the agronomic practices and production environments [24], dry matter content in sweet potato is genetically controlled [25–27], and has been shown to have direct influence on the starch content and *β*-carotene among other important root phytochemicals [28]. The need for breeding OFSP varieties with high dry matter content remains a critical issue in Kenya where most consumers prefer roots of high dry matter content. For instance, Kabode variety that is preferred by farmers and processors as an OFSP of choice in western Kenya had relatively a low dry matter content thus requiring improvement if Kabode has to be promoted for fried products. However, the variety with low root dry matter content may be suitable for slurry products such as puree.

### 3.2. Carotenoid Content

**Figure 2:**
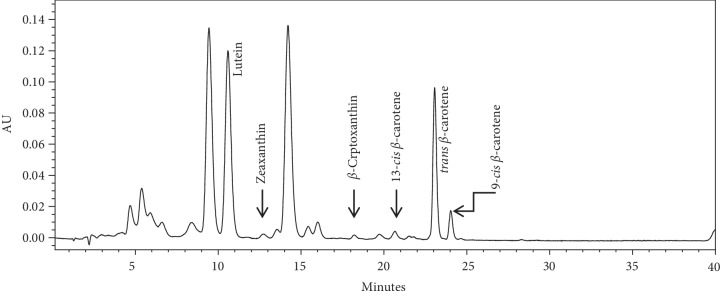
Carotenoids chromatogram for sweet potato leaves.

**Figure 3:**
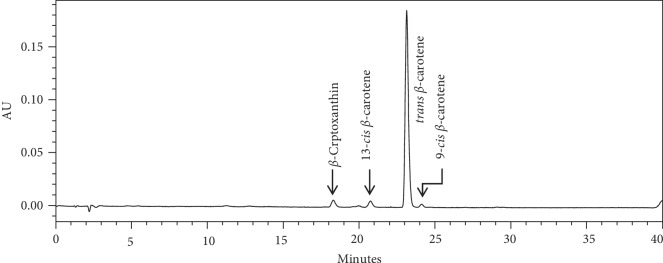
*β*-Carotene chromatogram for sweet potato roots.

Figures 2 and 3 illustrate HPLC chromatograms of major carotenoids and their variations in sweet potato leaves and roots. Total carotenoid content differed significantly (*p* < 0.05) among varieties and plant parts, the leaves indicating significantly (*p* < 0.05) higher values than the roots (Table 2). No carotenoid was detected in the local white variety. Lutein content was the highest (0.11 mg/100 g) in SPK031 roots, zeaxanthin was the highest (0.26 mg/100 g) in Kenspot 5, and *β*-cryptoxanthin was the highest (0.26 mg/100 g) in SPK4. All trans beta carotene was the highest (9.86 mg/100 g) in Vitaa, 13 cis *β*-carotene was the highest (0.39 mg/100 g) in SPK031 and 9 cis *β*-carotene was the highest (0.39 mg/100 g) in SPK031. In sweet potato leaves, lutein ranged 28.57–51.35 mg/100 g, zeaxanthin ranged 0.33–3.53 mg/100 g, *β*-cryptoxanthin ranged 0.28–0.65 mg/100 g and 13 cis *β*-carotene ranged 2.24–4.78 mg/100 g. All trans *β*-carotene ranged 13.33–28.07 mg/100 g while 9 cis *β*-carotene content ranged 2.24–4.78 mg/100 g.

Lutein was the most abundant carotenoid in sweet potato leaves while All trans *β*-carotene was the most abundant carotenoid present in the roots. The findings of this study were in agreement with general observation of previous research [29–31]. Carotenoids contribute towards root and fruit color, attractiveness, and quality parameters as well as play essential biological functions in humans with *β*-carotene and *β*-cryptoxanthin being important provitamin A, while lutein and zeaxanthin are natural antioxidants and important for eye health and cognition [32]. The content of *β*-carotene in the roots is comparable to 8.65 mg/100 g reported by Odongo et al. [33], 5.9–12.8 mg/100 g reported by Vimala et al. [34], and 0.38–7.38 mg/100 g reported by Alam et al. [35]. The *β*-carotene content in the leaves were, however, lower compared to an average of 53.32 mg/100 g for Tanzanian sweet potato varieties [36]. The lutein content was higher compared to the range of 19.01–28.85 mg/100 g reported in the same study. Carotenoids content in plants is influenced by genetics and cultural practices and hence vary between locations [5, 24].

### 3.3. Ascorbic Acid, Flavonoids, Total Phenolic Content and Antioxidant Activity

Variations in ascorbic acid, flavonoids and phenolic contents in leaves and roots of nine Kenyan sweet potato varieties are presented in Table 3. Vitamin C varied significantly (*p* < 0.05) among the sweet potato varieties regardless of the plant part. The leaves showed significantly (*p* < 0.05) higher vitamin C levels compared to the roots. The vitamin in roots ranged from 4.53 (Vitaa) to 19.05 mg/100 g (K/KA/2004/205) while in the leaves it ranged from 46.64 (Kenspot 5) to 349.05 mg/100 g (Vitaa).

Flavonoid and phenolic compounds differed significantly (*p* < 0.05) among varieties and with plant part, being higher in leaves than in roots. Flavonoids in roots ranged from not detectable (white fleshed) to 25.8 mg CE/100 g (K/KA/2004/215) while in the leaves it ranged from 4097 (SPK4) to 7316 mg CE/100 g (Kenspot 5). Phenolic content was not detected in white roots but was highest (224 mg GAE/100 g) in SPK031 roots. In the leaves, phenolics content ranged from 4496 to 6801 mg GAE/100 g in SPK031 and Kenspot 5, respectively. Antioxidant activity was significantly (*p* < 0.05) higher in leaves than in roots. It was the lowest (3827.3 mgTE/100 g) in K/KA/2004/215 and the highest (4707.6 mgTE/100 g) in SPK031 leaves while in the roots it ranged from 13.56 to 76.6 mgTE/100 g in SPK4 and K/KA/2004/215, respectively.

The vitamin C content in roots found in this study was comparable to 10 mg/100 g reported by [37], but was lower compared to Yildirim et al. [38] who reported a range of 23.7–38.6 mg/100 g. Compared to potato tubers [39, 40], the roots vitamin C content in tested sweet potato varieties was lower. The leaves exhibited high vitamic C content that are comparable to conventional fruits and leafy vegetables that range between 2 and 500 mg/100 g dry weight [41]. It is important to note that vitamin C is an essential vitamin for proper functioning of the human body to maintain redox balance, prevent the haemorrhagic disease scurvy, develop connective tissues, synthesise amino acids, and absorb iron in the gastrointestinal tract [42, 43]. The contribution of sweet potatoes to these functions depends on the form in which the products were consumed since ingested levels depend on processing method [44, 45].

The root phenolic contents in the current study were comparable to a range of 146–266 mg GAE/100 g reported for Australian Pindan Walnut [46], but lower than those reported for four coloured sweet potato varieties that ranged 960–5460 mg GAE/100 g [47]. This difference can be attributed to the fact that the analysis in the latter included the skins of the roots unlike in the current study where the skins were peeled as this is the common practice in Kenya. The current findings of high flavonoids and phenolics content in leaves than roots are in agreement with other studies [7]. Flavonoids and phenolic compounds have been shown to contribute towards high antioxidant properties that can contribute to prevention of diseases such as cardiovascular conditions and cancer [48]. They have also bee shown to improve the ability of the body to counteract oxidative stress in human dermal fibroblasts [49].

Antioxidant activity was significantly (*p* < 0.05) higher in leaves than in roots ranging from 3827 to 4708 mgTE/100 g and 13.56 to 79.6 mgTE/100 g, repsectively. The more coloured roots had higher antioxidant activity [50]. It is, however, noted that antioxidant activity does not depend solely on coloured compounds analyzed in this study since the white fleshed roots with undetectable levels of phenolics and flavonoids had considerable amounts of antioxidant activity. The sweet potato leaves with high amounts of phytochemicals indicated quite high levels of free radical scavenging activity, the highest correlation (*r* = 0.975, *p* < 0.0001) being displayed by total flavonoids (Table 4) showing the contribution of these phytochemicals to antioxidant activity [32, 39] as indicated by significant positive correlattion. High levels of phenolics, flavonoids, vitamin C and antioxidant properties in sweet potatoes, and especially, the leaves can therefore be exploited to prepare different food mixes with high antixidant properties useful in preventing and controlling some lifestyle diseases.

### 3.4. Antinutrient Factors

#### 3.4.1. Variations of Phytates

**Figure 4:**
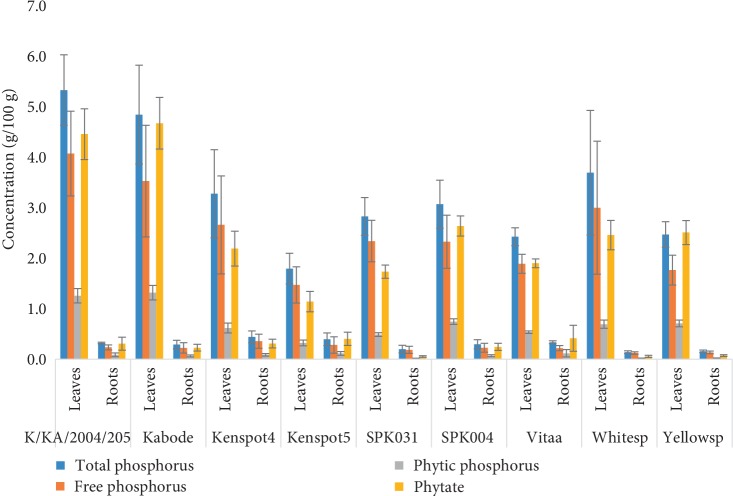
Variations in phosphorus and phytate contents in roots and leaves of nine Kenyan sweet potato varieties. The bars indicate standard error of the means.

Phytate was significantly (*p* < 0.05) higher in leaves than in roots. It was the lowest in Kenspot 5 (1.14 g/100 g) and the highest in Kabode leaves (5.33 g/100 g). In roots, phytate ranged from 0.05 to 0.42 g/100 g in SPK031 and Vitaa, respectively (Figure 4). Total and phytic phosphorus directly (*r* = 1, *p* < 0.05) correlated with phytate contents in both the leaves and the roots. Total and free phosphorus significantly (*r* = 0.976, *p* < 0.05) correlated with phytate content.

Phytates in plant foods vary with variety and plant part due to different genetic and physiological make up. Contrary to the findings of Dako et al. [51] who indicated that yellow varieties had higher phytates compared to white fleshed and orange fleshed sweet potato, in the present study yellow varieties contained phytate in moderate to lowest values. The current phytate values are slightly higher compared to their average of 0.05–0.08 g/100 g, probably due to the larger number of varieties included in this study. Lower values were also reported by Olapade and Ogunade [52] and Abubakar et al. [53]. These values especially in the leaves were, however, comparable to those (2.81–3.01 g/100 g) reported for cereals and other vegetables [54]. Phytic acid binds phosphorus in the food matrix. The findings of the present study showed high accumulation of phosphorus being linked to high phytate content, which means that high phytate varieties may also provide good amounts of phosphorus should processing mechanism significantly reduce phytates. The ratio of phytic phosphorus to total phosphorus in leaves was moderate (15–25%) and in agreement with values of 21–25 reported by Ravindra et al. [55]. However, phytic ratio of the roots had very high range (4–40%) and contrasted these earlier findings. The root phytic ratio were still lower than what Ravindran [55] reported for cereals and legumes, 60–70%, indicating that roots phytates accumulate more phosphorus.

#### 3.4.2. Variations in Tannins and Soluble Oxalates

Tannins varied significantly (*p* < 0.05) with variety and plant part being higher in leaves (40 times) than in roots (Table 5). Tannin contents in leaves ranged from 0.87 (Kabode) to 5.05 g/100 g (K/KA/2004/205), while in roots it ranged from 0.003 (Whitesp) to 0.132 g/100 g (SPK031). Root tannin values were not different from the average value (0.03 g/100 g) reported for yellow fleshed varieties by other researchers [51]. It is noted that tannin levels reported in this study are quite lower than levels (779–994%) reported in flours of sweet potato grain mixes [56]. Leaves' tannin content in this study was comparable to previously reported range of 2.28–4.46 g/100 g [57]. Tannins are complex plant metabolites that form part of polyphenols with considerably good medicinal properties. Tannins can, however, be regarded as antinutrients that bind essential minerals such as iron and significantly reduce their availability and hence the need to minimize them in most foods intended for mineral supplementation [20, 52, 58].

Oxalates were significantly (*p* < 0.05) higher in leaves than in roots. Within the plant portions, oxalates significantly (*p* < 0.05) varied with variety. Highest oxalate level (1618.7 mg/100 g) was recorded in leaves of Yellowsp varieties while the lowest (511.62 mg/100 g) was recorded in Kenspot 4. Oxalates in roots ranged from 25.58 to 235 mg/100 g in Vitaa and Kenspot 4, respectively. These values were extremely higher than the range of 5–12 mg/100 g reported earlier by Dako and others [51] and Olapade and Ogunade [52]. Roots values were, however, comparable to 126.9–178.3 mg/100 g range reported in an earlier study [53]. Leaves of all sweet potato varieties may not be suitable for frequent human consumption especially for those with kidney stones problems due to the high levels of oxalates exhibited in these varieties [51]. Oxalates bind calcium and magnesium and interfere with their absorption and metabolism hence the need to limit dietary intake. For proper utilization of leaves, therefore, appropriate processing mechanism is needed to reduce the tannins and oxalates.

## 4. Conclusion

For the first time, this study documents the phytochemical profiles of released Kenyan sweet potato varieties, as well as high variations among varieties for inherent phytochemicals. The leaves were superior in all aspects of phytochemicals that were evaluated. The information generated from this study is useful for ration formulations and dietary recommendations. Study on effects of processing methods on these phytochemicals would, however, give a better picture of the actual amounts being ingested by consumers utilizing different OFSP products.

## Figures and Tables

**Table 1:** Physical characteristics of leaves and roots of selected sweet potato varieties grown in Kenya.

Variety	Local name	Shape	Leaf colour	Mean leaf length (cm)	Shape	Skin colour	Flesh colour	Mean root length (cm)	Mean root diameter (cm)	Root maturity period (month)
Vitaa	Vitaa	Deep, five lobed leaf	Green	6.0	Obovate	Purple	Orange	21	20	4
Kabode	Kabode	Deep, five lobed leaf	Green	6.5	Elliptic	Purple	Deep orange	23	21	4
SPK4	Kakamega 4	Deep, five lobed leaf	Green	5.5	Elliptic	Purplish red	Orange	21	19	4
SPK031	SPK031	Deep, narrow five lobed leaf	Green	5.5	Obovate	Purple	Orange	11	24	4
Kenspot 5	Kenspot 5	Moderate, five lobed leaf	Green, purplish veins on the back	6.0	Elliptic	Purplish red	Orange	12	18	5-7
Kenspot 4	Kenspot 4	Reniform shaped, lateral lobed leaf	Green	6.0	Elliptic	Purplish red	Orange	15	18	5–7
Whitesp	Nyawoo	Moderate, five lobed leaf,	Green	5.5	Elliptic	Purple	White	25	24	6
Yellowsp	Nyamogo	Deep, five lobed leaf	Green	6.0	Elliptic	Cream	Yellow	25	23	6
K/KA/2004/215	Jankaroti	Deep, five lobed leaf	Young leaf-purplish, old leaf-green with purplish colour at the back	7.0	Elliptic	Cream	Light orange	16	23	4

Leaf and root parameters are mean values of 10 representative samples for each variety.

**Table 2:** Variations in carotenoids content (mg/100 g dry basis) among nine Kenyan sweet potato varieties.

Variety	Plant part	Lutein	Zeaxanthin	BX	13ZBC	AllTBC	9ZBC
K/KA/2004/215	Roots	0.10 ± 0.01^e^	0.10 ± 0.07^ g^	0.24 ± 0.10^ cd^	0.06 ± 0.01^i^	3.82 ± 0.07^f^	0.06 ± 0.01^f^
	Leaves	36.68 ± 2.74^c^	0.75 ± 0.00^bc^	0.39 ± 0.08^bc^	2.71 ± 0.24^d^	16.93 ± 2.44^ cd^	2.71 ± 0.24^ cd^
Kabode	Roots	0.02 ± 0.00^hg^	0.08 ± 0.02^ g^	0.18 ± 0.06^d^	0.06 ± 0.00^i^	4.65 ± 0.20^f^	0.06 ± 0.00^ g^
	Leaves	32.31 ± 0.35^ cd^	0.42 ± 0.18^de^	0.28 ± 0.05^ cd^	2.37 ± 0.11^e^	14.29 ± 0.30^d^	2.37 ± 0.11^d^
Kenspot 4	Roots	0.03 ± 0.00^ g^	0.02 ± 0.00^ h^	0.07 ± 0.01^e^	0.10 ± 0.04^ h^	2.64 ± 0.38^ g^	0.10 ± 0.06^f^
	Leaves	44.66 ± 1.88^b^	0.71 ± 0.04^bcd^	0.20 ± 0.01^ cd^	3.19 ± 0.21^bc^	19.49 ± 0.90^bc^	3.19 ± 0.21^bc^
Kenspot 5	Roots	0.10 ± 0.00^e^	0.26 ± 0.02^f^	0.46 ± 0.06^b^	0.12 ± 0.01^ h^	9.28 ± 0.10^e^	0.12 ± 0.01^ef^
	Leaves	28.57 ± 1.07^d^	0.60 ± 0.04^cde^	0.65 ± 0.24^a^	2.58 ± 0.21^de^	15.85 ± 0.53^ cd^	2.58 ± 0.21^d^
SPK031	Roots	0.02 ± 0.01^hg^	0.03 ± 0.02^ h^	0.45 ± 0.18^b^	0.39 ± 0.04^f^	18.18 ± 3.74^cb^	0.39 ± 0.04^e^
	Leaves	35.80 ± 2.52^c^	0.33 ± 0.01^e^	0.15 ± 0.01^de^	2.78 ± 0.17^d^	16.81 ± 1.38^ cd^	2.78 ± 0.17^ cd^
SPK4	Roots	0.11 ± 0.01^e^	0.24 ± 0.01^f^	0.26 ± 0.05^ cd^	0.05 ± 0.01^i^	4.43 ± 0.28^f^	0.05 ± 0.01^f^
	Leaves	32.35 ± 1.27^ cd^	0.52 ± 0.09^cde^	0.39 ± 0.04^bc^	3.32 ± 0.18^b^	21.15 ± 1.12^b^	3.32 ± 0.18^b^
Vitaa	Roots	0.01 ± 0.01^ h^	0.07 ± 0.01^ g^	0.35 ± 0.01^bc^	0.21 ± 0.01^ g^	9.86 ± 0.98^e^	0.21 ± 0.01^ef^
	Leaves	29.69 ± 0.14^d^	0.54 ± 0.06^cde^	0.26 ± 0.02^ cd^	2.24 ± 0.04^e^	13.33 ± 0.16^d^	2.24 ± 0.04^d^
Whitesp	Roots	ND	ND	ND	ND	ND	ND
	Leaves	48.28 ± 2.37^ab^	1.00 ± 0.01^c^	0.39 ± 0.02^bc^	4.43 ± 0.23^a^	27.37 ± 1.36^a^	4.43 ± 0.23^a^
Yellowsp	Roots	0.07 ± 0.03^f^	0.13 ± 0.03^ g^	0.07 ± 0.01^e^	0.03 ± 0.00^j^	0.73 ± 0.01^ g^	0.03 ± 0.00^f^
	Leaves	51.35 ± 4.4^a^	3.53 ± 0.33^a^	0.60 ± 0.01^ab^	4.78 ± 0.43^a^	28.07 ± 2.93^a^	4.78 ± 0.43^a^

BX = *β*-xanthin, 13ZBC = 13 Cis *β*-carotene, AllTBC = All trans *β*-carotene, 9ZBC = *β*-9 Cis *β*-carotene, ND = Not detected. Results are means of triplicate samples ± standard deviation; values with same letters in the superscript in the same column are not significantly different at *P* < 0.05.

**Table 3:** Ascorbic acid, flavonoid, phenolic and antioxidant contents (in dry weight basis) in leaves and roots of nine Kenyan sweet potato varieties.

Variety	Plant part	Ascorbic acid (mg/100 g)	Flavonoids (mgCE/100 g)	Phenolics (mgGAE/100 g)	Antioxidant (mgTE/100 g)
K/KA/2004/215	Leaves	192.28 ± 14.16^e^	6162.26 ± 236.60^c^	6134.03 ± 474.33^ab^	3827.30 ± 55.75^c^
	Roots	19.05 ± 1.25^ k^	25.85 ± 2.76^f^	190.50 ± 16.39^f^	79.60 ± 9.32^a^
Kenspot 4	Leaves	174.98 ± 8.5^e^	6247.64 ± 334.96^cb^	6313.33 ± 375.74^ab^	4074.35 ± 174.95^bc^
	Roots	4.95 ± 0.49^ l^	1.39 ± 0.00^i^	103.68 ± 8.00^ h^	27.67 ± 0.77^d^
Kenspot 5	Leaves	146.64 ± 0.09^f^	7315.83 ± 685.41^a^	6801.09 ± 325.38^a^	4222.82 ± 82.26^b^
	Roots	15.07 ± 1.34^ g^	1.03 ± 0.13^i^	139.79 ± 13.94^ g^	72.68 ± 7.63^b^
Kabode	Leaves	341.87 ± 23.74^a^	5975.55 ± 336.12^c^	5842.57 ± 233.69^bc^	4546.49 ± 348.07^a^
	Roots	16.56 ± 0.30^f^	21.81 ± 2.12^ g^	95.18 ± 8.74^ h^	41.41 ± 3.88^c^
SPK031	Leaves	297.45 ± 12.95^b^	4743.15 ± 420.31^d^	4495.93 ± 365.08^d^	4707.62 ± 187.05^a^
	Roots	11.06 ± 0.27^i^	25.81 ± 2.77^f^	223.55 ± 15.16^e^	39.52 ± 3.94^c^
SPK4	Leaves	272.29 ± 6.05^c^	4097.22 ± 384.84^e^	6432.73 ± 616.53^ab^	4055.10 ± 402.99^bc^
	Roots	13.33 ± 0.00^ h^	4.21 ± 0.17^ h^	92.75 ± 2.08^ h^	13.56 ± 1.01^e^
Vitaa	Leaves	349.05 ± 13.14^a^	6941.51 ± 211.24^a^	5749.26 ± 574.89^bc^	4027.98 ± 15.61^bc^
	Roots	4.53 ± 0.13^ l^	12.63 ± 0.00^ g^	176.72 ± 9.72^f^	31.86 ± 1.46^d^
Whitesp	Leaves	331.57 ± 5.77^a^	6868.09 ± 0.00^ab^	5216.71 ± 0.00^c^	4124.12 ± 266.90^bc^
	Roots	8.95 ± 0.52^j^	ND	ND	38.02 ± 2.97^c^
Yellowsp	Leaves	216.95 ± 11.54^d^	6868.09 ± 0.00^ab^	5216.71 ± 0.00^c^	4101.29 ± 354.34^bc^
	Roots	6.60 ± 0.65^ k^	12.63 ± 0.00^ g^	187.42 ± 0.00^f^	75.17 ± 8.88^ab^

ND = Not detected; results are means of triplicate samples ± standard deviation; Values with similar letters in the same column are not significantly different at *P* < 0.05.

**Table 4:** Pearson correlation (*r*) between phytochemicals and antioxidant property.

Parameter	Ascorbic acid	Flavonoids	Phenolics	Total carotenoids	*β*-Carotene
Antioxidant property	0.931	0.964	0.975	0.923	0.831
*p* value	<0.0001	<0.0001	<0.0001	<0.0001	<0.0001

**Table 5:** Tannins and soluble oxalates (dry basis) in leaves and roots of nine Kenyan sweet potato varieties.

Variety	Plant part	Oxalates (mg/100 g)	Tannins (g/100 g)
K/KA/2004/215	Leaves	853.83 ± 26.55^c^	5.05 ± 0.05^a^
	Roots	130.58 ± 4.88^ij^	0.09 ± 0.01^ g^
Kenspot 5	Leaves	687.93 ± 59.77^d^	4.22 ± 0.33^bc^
	Roots	152.52 ± 23.47^i^	0.07 ± 0.00^ h^
SPK004	Leaves	827.34 ± 52.59^c^	4.51 ± 0.12^b^
	Roots	87.21 ± 1.22^ l^	0.05 ± 0.01^i^
Vitaa	Leaves	657.38 ± 112.72^de^	4.53 ± 0.17^b^
	Roots	25.58 ± 1.45^ m^	0.08 ± 0.01^ h^
WhiteSP	Leaves	796.87 ± 9.73^c^	3.84 ± 0.23^ cd^
	Roots	98.62 ± 13.43^kl^	0.04 ± 0.00^i^
Kabode	Leaves	1369.09 ± 81.47^b^	0.87 ± 0.11^j^
	Roots	793.31 ± 40.03^c^	0.10 ± 0.01^ g^
Kenspot 4	Leaves	511.62 ± 20.54^f^	4.00 ± 0.39^b^
	Roots	235.01 ± 48.73^ g^	0.04 ± 0.00^i^
SPK031	Leaves	741.34 ± 87.07^c^	3.57 ± 0.53^d^
	Roots	180.98 ± 10.96^ h^	0.13 ± 0.02^f^
Yellowsp	Leaves	1618.71 ± 42.39^a^	2.91 ± 0.14^e^
	Roots	122.83 ± 6.69^j^	0.07 ± 0.00^ h^

Results are means of triplicate samples ± standard deviation; values with similar letters in the same column are not significantly different at *P* ≤ 0.05.

## Data Availability

The data used to support the findings of this study are available from the corresponding authors on request.
